# Blue dye degradation in an aqueous medium by a combined photocatalytic and bacterial biodegradation process

**DOI:** 10.3906/kim-1902-33

**Published:** 2020-02-11

**Authors:** Gabriela PÉREZ-OSORIO, Flor del Rocío HERNÁNDEZ-GÓMEZ, Janette ARRIOLA-MORALES, Maribel CASTILLO-MORALES, José Carlos MENDOZA-HERNÁNDEZ

**Affiliations:** 1 Faculty of Chemical Engineering, Benemérita Universidad Autónoma de Puebla, Puebla México

**Keywords:** Water pollution, dyes, photocatalysis, solar radiation, bacterial degradation, toxicity

## Abstract

This paper aimed at implementing a treatment system for polluted water with textile dyes, starting with a photocatalytic decomposition process using sunlight as a source of energy and continuing with a bacterial biodegradation process, in order to reach degradation percentages higher than those obtained using only one of the processes mentioned above. When water treatment with the dye in the combined system was over, an acute ecotoxicity test was performed to make sure that toxic metabolites were not produced due to biodegradation. Solophenyl Blue azoic dye, and Erionyl Blue and Terasil Blue anthraquinone dye-colored solutions were treated with the Pd/Al _80_ Ce _10_ Zr _10_ catalyst in a solar collector for the photocatalytic process. On the other hand, the waste dye, which was obtained from photocatalysis with a bacterial consortium from polluted areas by metals and hydrocarbons in aerobic conditions, was inoculated for biodegradation. Biodegradation was obtained for the dyes after both processes as 90.91% for the Solophenyl Blue azoic dye, and 87.80% and 87.94%, respectively, for the Erionyl Blue and Terasil Blue anthraquinone dyes. After the degradation processes, it was proven, via an ecotoxicity test with *Daphnia magna* , that toxic metabolites had not been produced.

## 1. Introduction

Worldwide, 280,000 tons of textile dyes are discharged into industrial effluents every year [1]. Although, dyes are widely used in the cosmetic, pharmaceutical, food, plastics, printing, and leather industries [2], the main generator of contaminated effluents with dyes is the textile industry, since it represents two-thirds of the total colorant market [3,4]. Azo dyes represent most of the textile dyes produced due to the facility and economics of their synthesis, and their stability and variety of available colors when compared with natural dyes [5]. Anthraquinone dyes are the second most important chemical class of dyes, and the main reason why they have less commercial importance than Azoic dyes is their high cost [6,7].

The discharge of contaminated effluents by dyes generates negative effects in aquatic ecosystems, among which are a decrease in the concentration of dissolved oxygen and penetration of sunlight [2], inhibited microorganism growth, adverse affects on aquatic flora [8], and their carcinogenicity in humans [9]. The most common chemical methods for the treatment of textile effluents include coagulation/flocculation; filtration; advanced oxidation processes (AOPs); sonolysis techniques and ultraviolet photolysis; nano-, micro-, and ultrafiltration; and reverse osmosis [10,11]. The above-mentioned methods have disadvantages that hinder their application, because some are unable to completely remove the azo dyes and/or their metabolites due to their stability and resistance during the degradation processes [5], thus generating toxic products [5,10,12]. AOPs have gained importance in the area of wastewater treatment as an emerging destructive technology [13] used as pretreatments for biological techniques because they generate biocompatible effluents [14]. In AOPs, the catalyst plays a major role, with TiO _2_ being the most used and efficient to degrade organic recalcitrant compounds due to its low cost, inert nature, and photostability. However, this catalyst has some disadvantages, such as the generation of turbidity, use of high doses, and need to use UV radiation. The current research has focused on proposing alternative materials, such as either TiO _2_ doped with Ag, Ce, or V, or other semiconductors such as ZrO _2_ , ZnO, or materials with different characteristics, but with the ability to promote oxidation and reduction reactions on surfaces. It is also aimed that these alternative materials will have a band gap lower than that of the TiO _2_ to achieve reactions with the incidence of solar radiation [15,16]. On the other hand, biotechnological treatments, with the use of fungus, yeasts, and mainly bacteria, are more frequently used in the elimination of dyes because they are environmentally friendly technologies [3,10,12].

Because many of the techniques for the removal of dyes can generate toxic byproducts, it is important to consider conducting a toxicity test on the products of these treatments to ensure that their release into the environment will not cause side effects [1,5]. One of the most common ecotoxicological tests of sweet-water effluents is with *Daphnia magna* [17]. This organism is the most commonly used bioindicator in toxicity tests, mainly due to its wide geographic distribution, ease of handling, low operating costs, and sensitivity to the presence of toxic compounds [17,18].

In this research, the use of the catalyst Pd/Al _80_ Ce _10_ Zr _10_ , with a common formulation in catalytic conversion systems in automobiles, was proposed due to its ability to perform simultaneous reactions of oxidation and reduction, and eliminate the pollutants produced in combustion processes [19]. Recently, it has been applied for the elimination of organic compounds, such as benzene and toluene in aqueous medium [20,21]. In addition, palladium-supported catalysts increase efficiency in photocatalytic processes, since metal nobles facilitate the electron transfer from the conduction band to the water molecule [22].

## 2. Methodology

### 2.1. Photocatalytic decomposition

#### 2.1.1. Catalyst production and characterization

The catalyst support (Al _80_ Ce _10_ Zr _10_ ) was synthesized beforehand using the sol–gel method and later impregnated with palladium (0.2 wt%) [23]. A solution of cerium acetylacetonate (Sigma-Aldrich, St. Louis, MO, USA) and/or zirconium acetylacetonate (Alfa Aesar, Thermo Fisher Scientific, Lancashire, UK) in ethanol with moderate agitation was added to a mixture of aluminum sec-butoxide and 2-metil-2,4-pentanediol (Alpha Aesar), and allowed to reflux for 3 h, with moderate agitation at 94 ◦ C. Hydrolysis was performed by adding deionized water, drop-by-drop. The obtained gel was aged for 10 h. Samples were dried in a vacuum (10 ^-2^ Torr) at 100 ◦ C for 12 h, and then calcined in a N _2_ atmosphere at 450 ◦ C for 12 h, with a later treatment in air at 650 ◦ C for 4 h. After impregnation, the samples were again calcined in air at 650 ◦ C for 4 h. Physicochemical characterization of the catalyst, to determine the crystal structure, particle morphology, and chemical composition of the material, was performed using a Bruker model D8 Discover X-ray diffractometer (Billerica, MA, USA) and energy dispersive spectroscopy was performed using a JEOL JSM-6610 LV microscope (Tokyo, Japan) equipped with an Oxford INCA detector (Abingdon, UK).

The surface area was determined by the physisorption of N _2_ (–196 ◦ C) in a fully automatized Micromeritics ASAP 2020 volumetric absorption system (Atlanta, GA, USA). The isotherms of absorption–desorption were determined in the interval of P/Po 0.002 a 0.991. The catalyst was degassed with thermal treatments at 150 ◦ C over 3 h under low pressure. The Brunauer-Emmett-Taller (BET) equation was applied in the interval of linearity of the isotherm of absorption at relative pressure values (P/Po) of 0.05 to 0.3.

#### 2.1.2. Photocatalytic degradation by solar radiation

This analysis was performed in a tube solar collector (36 mm in external diameter, 1500 mm in length, and 1.4 L in volume) made of borosilicate, which had a 95% UV transmission on high reflectance aluminum sheets (involute) coupled in an air and refrigeration system to maintain air diffusion (1800 cc/min, ELITE 801 air pump; Hagen Inc., Baie-D’Urfe, Canada) and dispersion catalysts, to prevent evaporation of the water. Next, Terasil Blue, Solophenyl Blue, and Erionyl Blue were each prepared in a dye solution of 50 ppm for use in the degradation. Table 1 presents the characteristics and chemical formulas of the blue dyes used. The solutions were then added to 0.04 g of catalyst Pd/Al _80_ Ce _10_ Zr _10_ and exposed to solar radiation for 5 h. The solar collector was placed in an area where sunlight was received completely in a north–south orientation, with 19◦ of inclination (altitude of Puebla). Experiments were performed in September, beginning at 0900 hours for 5 h, and an aliquot of 10 mL was collected each hour with a UV-Vis spectrophotometer to evaluate dye degradation. At the end of the photocatalytic decomposition process, the total sample was collected and maintained at room temperature in the dark.

**Table 1:** Characteristics and the chemical formula of the blue dyes used.

Dye	Classification	Name	Chemical formula
Terasil Blue	Anthraquinone	Disperse Blue 56	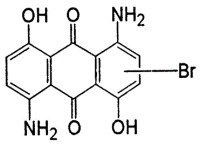
Erionyl Blue	Anthraquinone	Acid Blue ARL	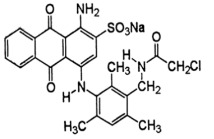
Solophenyl Blue	Azo	Direct Blue 85	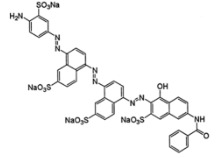

### 2.2. Bacterial biodegradation

#### 2.2.1. Bacterial strains

A consortium composed of 3 bacterial strains was used: *Serratia* MC107 and *Klebsiella* MC173, which were previously isolated from mine tailings and characterized as vegetable growth promotors [24] and *Pseudomonas putida* B44, which was isolated from a hydrocarbon spilling area in Acatzingo, Puebla, Mexico [25].

#### 2.2.2. Preparation of the inoculant

The inoculant for the biodegradation of the photochemical degradation waste dye was prepared by individually growing bacterial strains *Serratia* MC107, *Klebsiella* MC173, and *Pseudomonas putida* B44 in Luria Bertani broth, which was then incubated at 30 ◦ C for 48 h by orbital stirring at 80 rpm. Later on, they were separated by centrifugation at 8000 rpm for 20 min.

#### 2.2.3. Batch culture

A 5-L Tecno-lab MAG biological reactor was prepared containing 3 L of the photochemical dye reaction product to be degraded, and 12 g of the bacterial consortium, containing 4 g of each strain and micronutrients (composition per g L−1 : KH _2_ PO _4_ 0.4, K _2_ HPO _4_ 2, MgSO _4_ 0.2, CaCl _2_ 0.1, FeSO _4_ 0.005, H _3_ BO _3_ 0.002, ZnSO _4_ 0.005, NaMo 0.001, MnSO _4_ 0.003, CoSO _4_ 0.001, CuSO _4_ 0.001, NiSO _4_ 0.001) [24]. It was then incubated at 30 ◦ C by stirring at 80 rpm for 6 days.

The degradation percentage was monitored by taking 3 mL aliquots every 24 h for 6 days, and separating the bacteria by centrifugation at 8000 rpm for 10 min. The dye concentration was determined by UV-Vis spectroscopy in the supernatant, taking the maximum absorption band as a reference at 569 nm for Solophenyl Blue, 590 nm for Erionyl Blue, and 559 nm for Terasil Blue.

### 2.3. Kinetics of degradation

To determine the speed and factors of the chemical reaction that such reactions depend on during dye biodegradation, the reaction constant was defined by Eqs. (1)–(3), which are shown below [26]:

(1)CT=k0t+C0(2)CT=C0e-k1t(3)CT-1=-k2t+C0-1

where *C _T_* is the dye concentration at a certain time in ppm, C _0_ is the zero degree constant during degradationin ppm/h, k _1_ is the first degree constant during degradation in (h ^-1^ ), and k _2_ is the second degree constant during degradation in ppm/h.

Table 2 shows the experimentally obtained data for the determination of the kinetic constant degree (k) in the degradation of the 3 dyes. For the zero degree constant (k _0_ ), the concentration against time was considered; for the first degree constant (k _1_ ), the natural concentration logarithm against time was considered; and for the second degree constant (k _2_ ), the inverse concentration against time was considered. After that, the correlation quotient (R ^2^ ), slope (m), and origin ordinate (b) of the tabulated data were determined.

**Table 2:** Kinetic parameters obtained for the 3 degraded dyes.

	k _0_	R ^2^	k _1_	R ^2^	k _2_	R ^2^
Solophenyl Blue	–0.342705	0.903527	–0.017278	0.951484	0.001305	0.618448
Erionyl Blue	–0.127172	0.440363	–0.007689	0.983107	0.000573	0.916901
Terasil Blue	–0.159309	0.433724	–0.008590	0.987375	0.000602	0.922484

### 2.4. Infrared spectrum analysis

Simple absorbance before and after the degradation processes was analyzed by FTIR using Spectrum-One equipment from PerkinElmer (Waltham, MA, USA). The obtained spectrum ranged from 400 to 4000 cm ^-1^ . A minimal mineral medium was used for the equipment background and then the experimental sample spectra were obtained.

### 2.5. Acute toxicity test with *Daphnia magna*

The acute toxicity test of the degradation products from the 3 dyes, obtained at the end of the combined treatment system, was performed using the methodology stated in Norma Official Mexicana NMX-087-SCFI-2010 for the analysis of acute toxicity in water. Exploratory tests were done with 100%, 50%, 25%, 12.5%, and 6.25% of the samples obtained once the bacterial biodegradation ended, using reconstituted water as a dilution medium and, in the case of toxicity in the samples, the lethal dose 50 (LD50) was calculated by defining trials exposing the organisms to at least 5 dilutions in the range of observed toxicity. Every liter of reconstituted water was prepared with solutions of 25 mL of dehydrated calcium chloride (11.76 g), magnesium sulfate pentahydrate (4.93 g), sodium bicarbonate (2.59 g), and potassium chloride (0.23 g), all of which were diluted in 1 L. Placed into each sample container was 30 mL of each of the prepared diluted solutions and 10 newborns less than 24 h old. In each case, the trials were performed in triplicate for each dilution. Additionally, both a positive and a negative control were prepared to test the sensitivity and appropriate health status of the sample organisms, respectively. Exposure was 48 h in all cases, with prereadings at 24 h. These control readings allowed the presence or absence of movement in the reference organisms to be tested.

## 3. Results

### 3.1. Photocatalytic degradation

#### 3.1.1. Catalyst support structure

**Figure 1:**
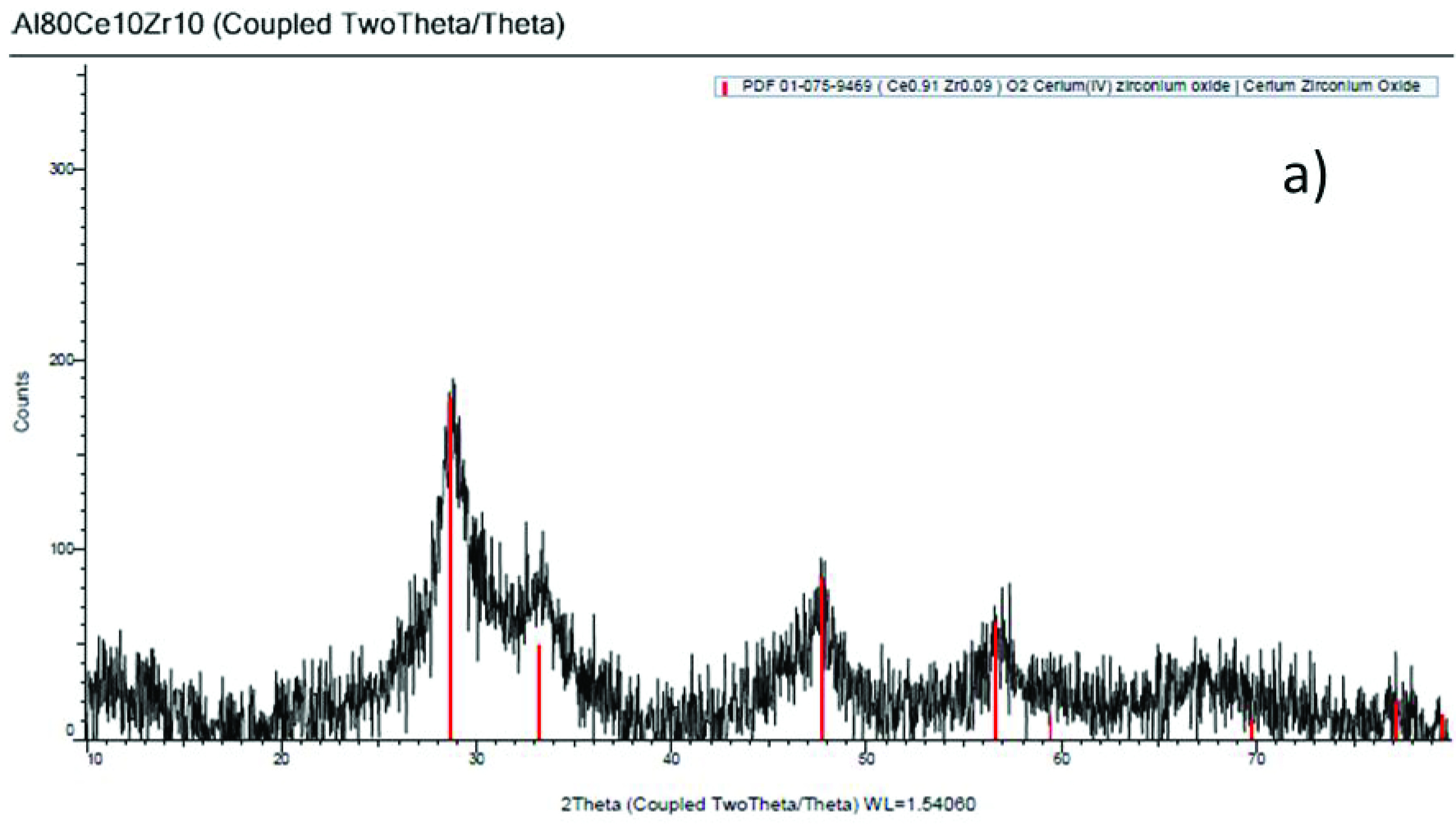

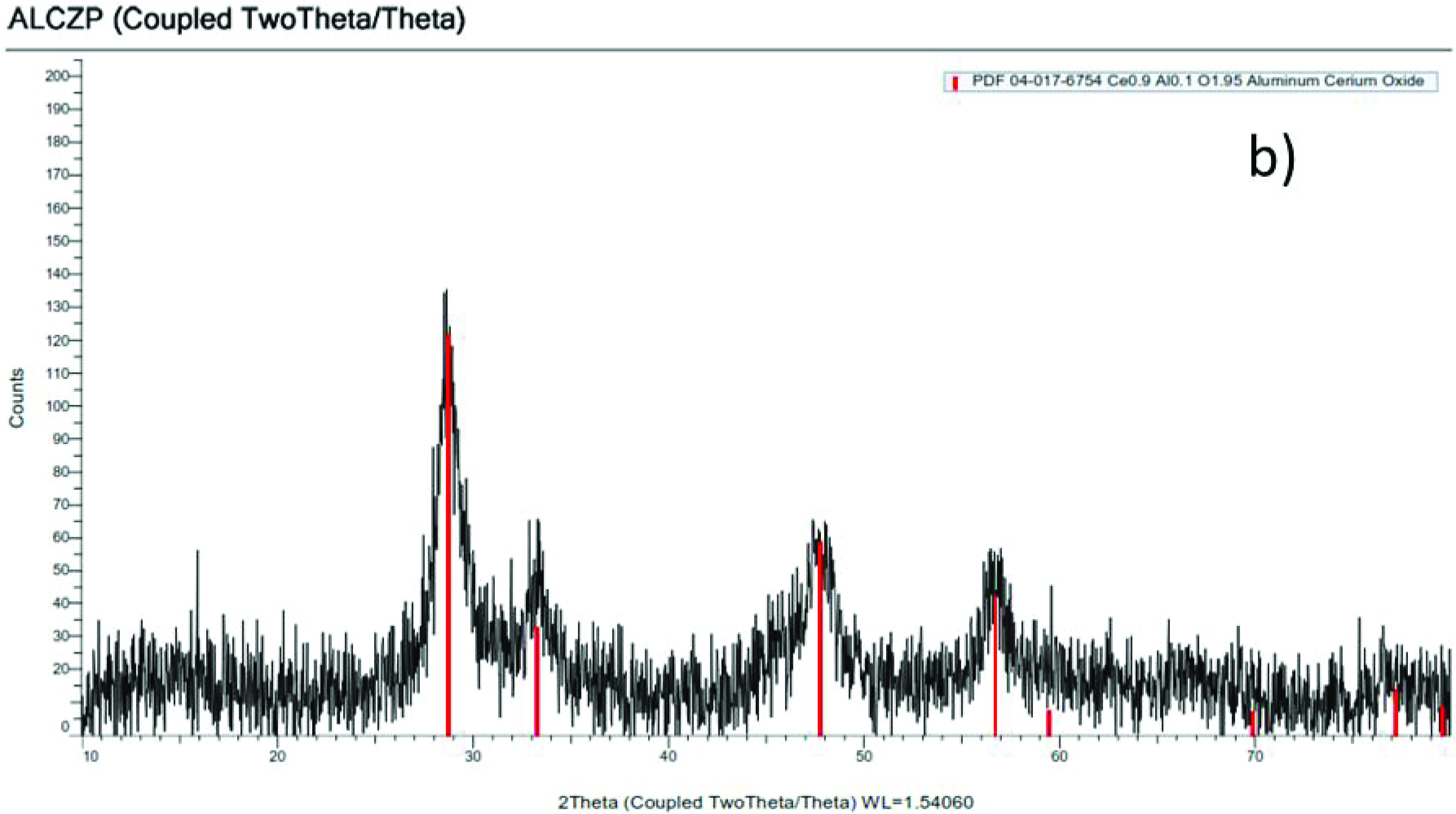
(a) X-ray diffraction pattern of the catalyst support Al _80_ Ce _10_ Zr _10_ . (b) X-ray diffraction pattern of the catalyst Pd/Al _80_ Ce _10_ Zr _10_ .

In the diffractogram of the catalyst support Al _80_ Ce _10_ Zr _10_ , (Figure 1a), wide peaks with scarce definition that showed the prevalence of amorphous areas, identified according to the database card 01-075-9469 [27], and cerium-zirconium oxide (Ce _0.91_ Zr _0.09_ )O _2_ , with a cubic shape and the 4 main signals at angles 2? of 28.6◦ , 33.2◦ , 47.7◦ , 56.6◦ can be observed. Signals belonging to aluminum oxide were not observed, although it had the highest percentage in the support composition. This occurrence was due to the fact that it is a completely amorphous material, and it can be assumed that on its surface, combined cerium and zirconium oxide particles that were able to crystallize in some areas had been deposited. Figure 1b, corresponds to the catalyst diffractogram, where palladium was not identified because the concentration was below the detection limit of the equipment used. In general, the addition of palladium does not produce structural changes.

#### 3.1.2. Morphology and elemental chemical composition of the catalyst

**Figure 2:**
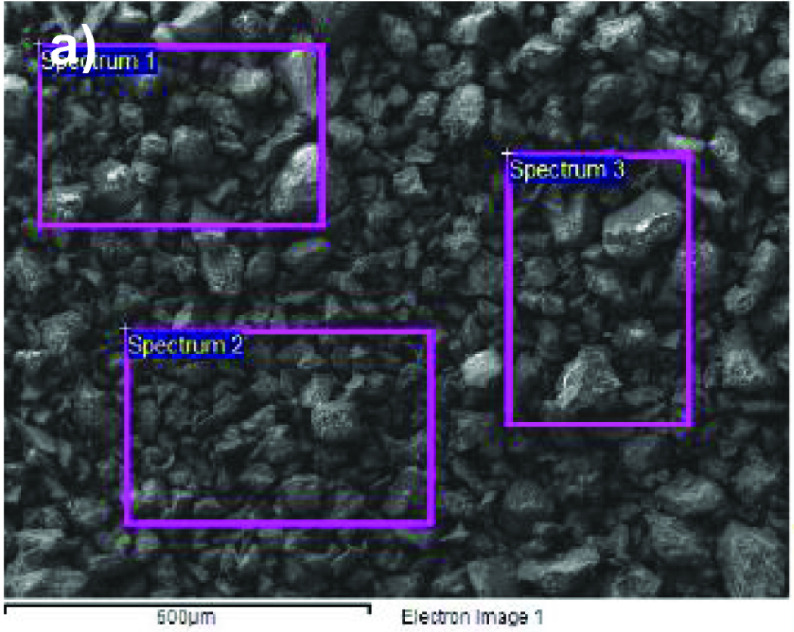

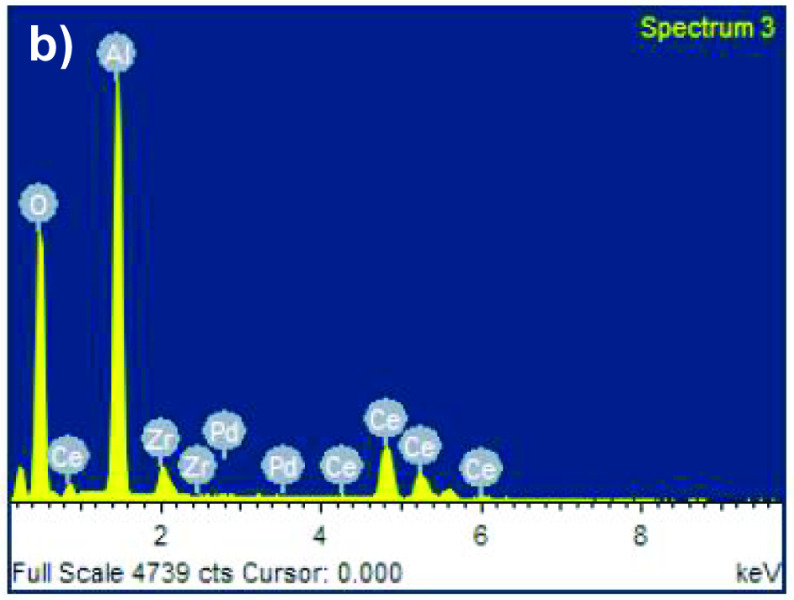
(a) Image of the SEM of the catalyst Pd/Al _80_ Ce _10_ Zr _10_ with a 50× magnification showing the 3 sampling areas for the EDS analysis. (b) X-ray EDS in area 3, obtained with a JEOL JSM 6610 SEM equipped with an OXFORD secondary electron detector.

The elemental chemical composition of the catalyst used was obtained via this analysis. In order to do so, 3 measurements were taken at random positions to obtain a composition average. Figure 2a shows an image of the catalyst Pd/Al _80_ Ce _10_ Zr _10_ obtained with the SEM at a magnification of 50X, where the particles are polymorphous and vary in size. Three measurement areas are shown, marked as Spectrums 1, 2, and 3, and from each one, an energy dispersive spectroscopy (EDS) spectrum with the elemental chemical composition was obtained, as with that in area 3, shown in Figure 2b. The average of the weight percentage (Table 3) of the present elements was O 46.51, Al 27.13, Zr 5.5, Pd 0.2, and Ce 20.67, so it was proven that a material with the desired characteristics and no impurities or residues in the precursors used was obtained by the sol-gel synthesis process. Unlike the analysis carried out by X-ray diffraction, it was confirmed here that aluminum oxide had the highest content in the catalyst, and that the palladium was dispersed in the support at a concentration of 0.2 wt%. The surface area BET was 131.2 m ^2^ g ^-1^ .

**Table 3:** Elemental chemical composition of the catalyst, obtained by EDS.

Spectrum	O	Al	Zr	Pd	Ce	Total
1	47.52	27.00	5.37	0.20	19.91	100
2	45.23	27.28	5.61	0.35	21.52	100
3	46.77	27.11	5.51	0.05	20.57	100
Mean	46.51	27.13	5.50	0.20	20.67	100
Standard deviation	1.17	0.14	0.12	0.15	0.81	

#### 3.1.3. Evaluation of dye concentration reduction by photocatalysis

**Figure 3:**
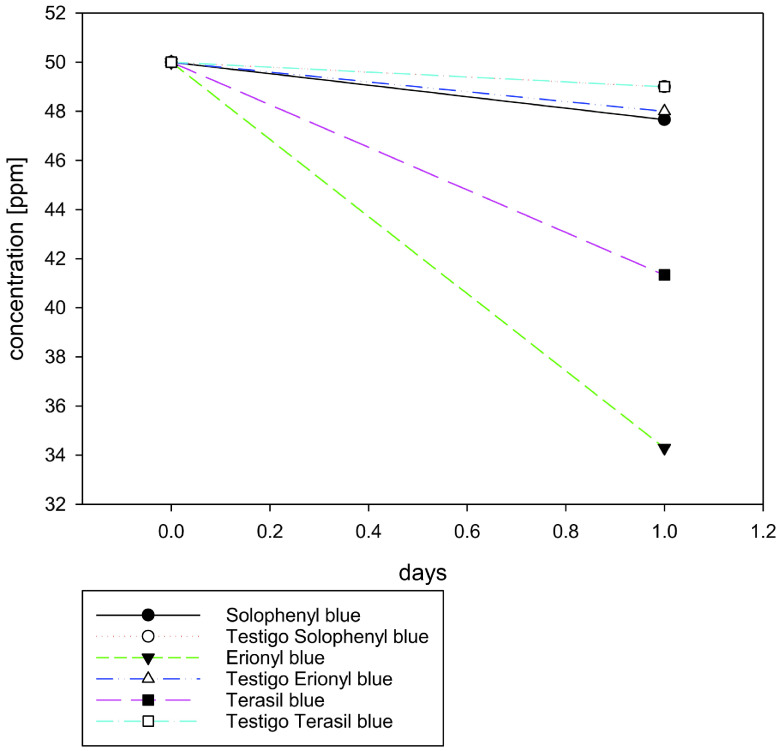

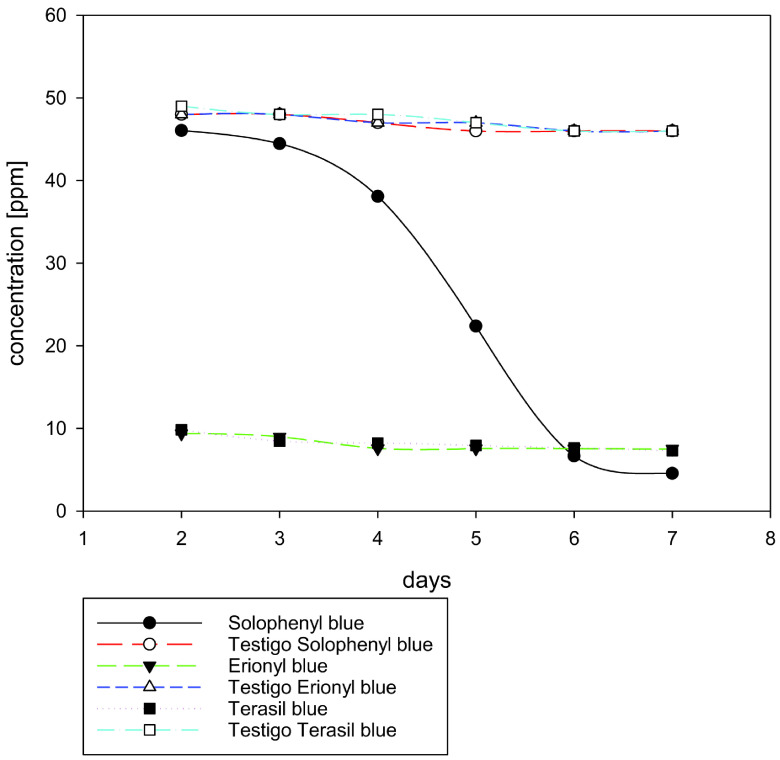
(a) Evaluation of the dye concentration reduction after applying the photocatalytic processes. (b) Evaluation of the dye concentration reduction after applying the bacterial processes.

The following results (Figure 3a) were obtained by simple UV-Vis spectroscopy after 5 h of exposure to solar radiation and contact with the catalyst, Pd/Al _80_ Ce _10_ Zr _10_ . With regards to the Solophenyl Blue azoic dye, 4.68% degradation was determined. On the other hand, the anthraquinone dyes had a higher degradation percentage, with the Terasil Blue dye at 17.31% and the Erionyl Blue dye at 31.42%. The pH of the samples was constant during treatment, at a value of 6 for the azoic dye and 5 for the anthraquinone dyes. Although the percentages were relatively low by this process, it is important to highlight that the catalyst was able to start the complex molecule destabilization more easily for the anthraquinone dyes than for the azoic dye, in only 5 h of exposure to solar radiation. The proposed catalyst had a structure and characteristics different from that of TiO _2_ , but it seemed to act as an indicator of oxidation reactions that allowed the degradation of the dyes under analysis. The combined oxide phase of cerium and zirconium is known as an efficient catalyst in oxidation reactions [28], whereas palladium is an oxidation and reduction reaction promoter when dispersed on the surface in the presence of accepting or donor electron molecules that have been formed, both by incident solar radiation and by adsorption phenomena that can be present on the catalyst surface and bulk [29].

### 3.2. Bacterial biodegradation

#### 3.2.1. Evaluation of dye concentration by UV-Vis spectroscopy

In contrast to the physicochemical treatment, the biodegradation process after the photocatalytic process had more effective results with the Solophenyl Blue azoic dye, where degradation was 86.23%, whereas it was lower with the Erionyl Blue and Terasil Blue anthraquinone dyes, with degradation of 43.49% and 56.16%, respectively (Figure 3b). The pH of the samples was constant during treatment at a value of 7 for all 3 dyes.

#### 3.2.2. Evaluation of dye concentration by UV-Vis spectroscopy after photocatalysis and bacterial biodegradation

At 7 days after the photocatalysis and bacteriological processes, biodegradation was 90.91% for Solophenyl Blue, 87.94% for Terasil Blue, and 87.80% for Erionyl Blue.

#### 3.2.3. Kinetics of degradation

With regards to heterogeneous catalytic reactions that developed on the surfaces, it is common to have zero order reactions. However, in this paper, the reactions were influenced and directed by other factors when applying 2 consecutive degradation processes (photocatalytic and bacterial). The kinetic constant was of first order for the degradation of the 3 dyes, which showed that the reaction depended only on the dye concentration present during the experiment. It must be stated that the negative value of the constant was due to the dye being consumed by the bacterial consortium.

### 3.3. Infrared spectrum analysis

**Figure 4:**
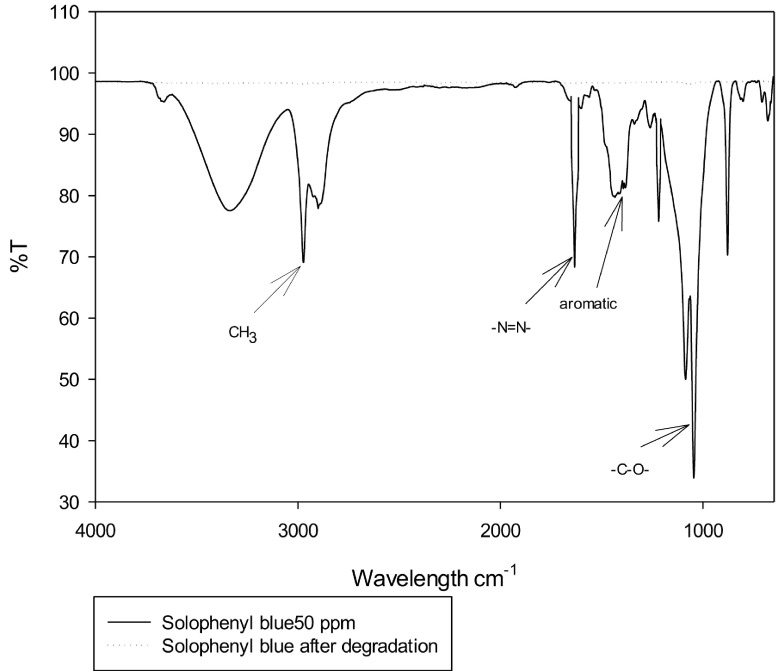

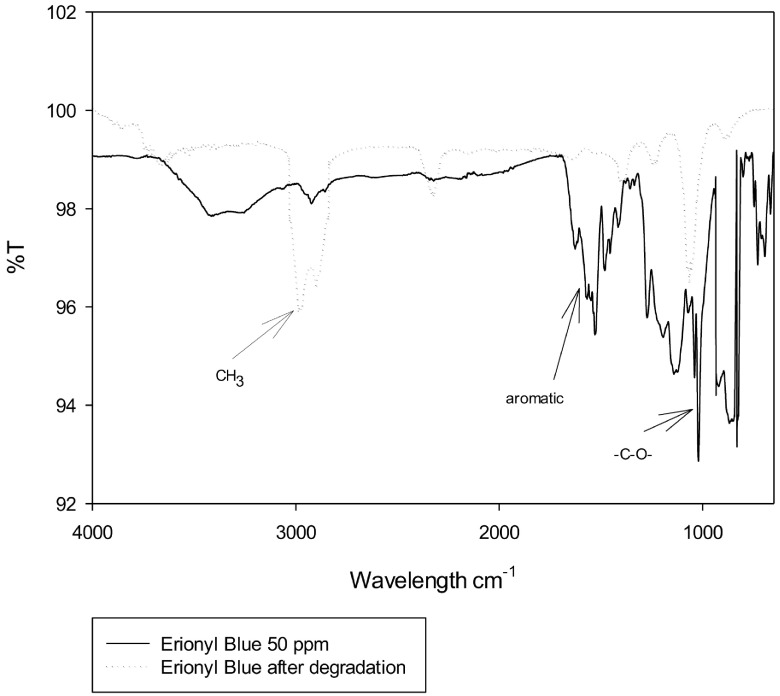

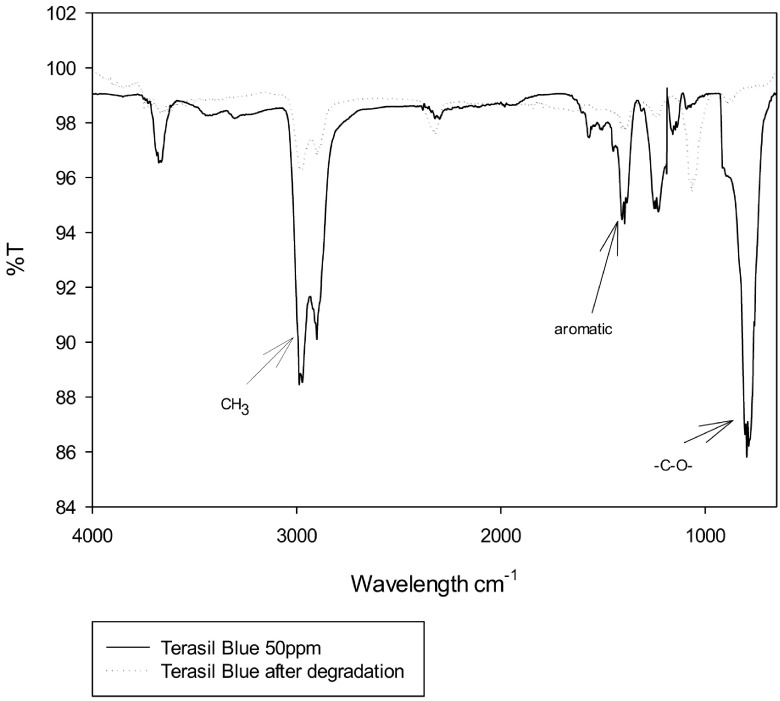
(a) IR spectrum of the Solophenyl Blue azoic dye, before and after the photocatalytic and bacterial degradation processes. (b) IR spectrum of the Erionyl Blue anthraquinone dye, before and after the photocatalytic and bacterial degradation processes. (c) IR spectrum of the Terasil Blue anthraquinone dye, before and after the photocatalytic and bacterial degradation processes.

Figure 4a shows the infrared spectrum of the Solophenyl Blue dye in a solution, which shows that the stretching vibration of the azo group –N = N– was seen at 1634 cm ^-1^ . The peaks at 1219 and 1045 cm ^-1^ were typical of the SO ^3-^ and –C–O–, respectively. The peaks at 2800–3100 cm ^-1^ were related to the vibrations of the linkage –CH, whereas the peaks at 1416–1522 cm ^-1^ belonged to the aromatic compounds. After the photocatalytic and bacterial processes, a reduction in the bands belonging to the azo group and the aromatic compounds could be observed, where the peaks increased in the region of 2800–3100 cm ^-1^ , which was typical of the linkage –CH. Hence, it can be said that a process of transformation from the dye to simpler carbonated compounds occurred. Peaks ranging from 670 to 870 cm ^-1^ were found in the anthraquinone dyes, which were due to the presence of aromatic rings that considerably decreased at the end of the decolorization process in the Erionyl Blue (Figure 4b) and Terasil Blue (Figure 4c) dyes, where there were groups in the regions of 2800–3100 cm ^-1^ that were typical of the linkage –CH. Thus, it showed that there was a transformation process of the dyes.

### 3.4. Acute toxicity test

The results obtained from the biotests with *Daphnia magna* for the analysis of the LD50 for Solophenyl Blue, Erionyl Blue, and Terasil Blue at the end of the combined process showed the absence of toxicity for this aquatic arthropod once 48 h of exposure had elapsed. Hence, it can be assumed that degradation occurred without generating any secondary toxic products with regards to each of the 3 dyes.

## 4. Discussion of the results

Chemical processes have been widely used for azo dye degradation, among which there is photocatalysis, Fenton’s reagent, chemical oxidation, ozonification, and nanoclusters [30,31]. In recent years, water purification processes by heterogeneous photocatalysis have become important because of degradation effectiveness or organic compound mineralization using sunlight or UV radiation as a source of energy, and TiO _2_ as a catalyst, which is nontoxic but is needed in high doses, resulting in turbidity, and is efficient only with UV radiation. As a result, this paper is important because it uses solar energy and looks for new catalysts that decrease the cost of the process and the formation of sludge.

In the photocatalytic process with the catalyst Pd/Al _80_ Ce _10_ Zr _10_ , degradation was 4.68% for the Solophenyl Blue azo dye, and 17.31% and 31.42% for the Terasil Blue and Erionyl Blue anthraquinone dyes, respectively, which were lower results than those reported in the literature [32,33], where the TiO _2_ catalyst and UV radiation (from mercury lamps) were used, obtaining higher degradation percentages (80%–100%) for the textile dye Procion yellow H-EXL, or via the ozonification process in anthraquinone dyes [34], but with a more expensive implementation cost. In this regard, the proposed catalyst Pd/Al _80_ Ce _10_ Zr _10_ has the advantage of working in the presence of sunlight, and it does not cause turbidity due to the low concentration used in comparison with titanium dioxide, though it is less effective in dye degradation. In addition to the photocatalytic degradation obtained, the initial phase of molecule destabilization of the dyes was due to the oxidation and reduction properties of the palladium catalyst. The higher level of influence in the anthraquinone dyes than in the azo dyes was remarkable because it showed that the latter dyes had a more resistant chemical structure to photochemical degradation processes.

Solophenyl Blue dye has the heaviest molecular weight of the 3 studied dyes and it has 3 azo groups in its structure, so it is highly resistant to degradation processes, whereas Terasil Blue and Erionyl Blue have a molecular weight lower than half that of Solophenyl Blue, and an anthraquinone group in their structures with substituting electron donors that allow the molecule to destabilize for degradation in the presence of highly oxidizing substances, such as the •OH groups formed during the photocatalytic processes. These titanium oxide processes are also used as pretreatment systems for dye decolorization [35].

Bacteria have been used for azo and anthraquinone dye decolorization, such as *Bacillus sp.* [36], *Salmonella sp.* , *Pseudomonas sp.* , *Pseudomonas putida* [37–39], and *Bacillus cereus* [40], under specific pH (ranging from 7 to 9.5) and temperature conditions, with a source of carbon and nitrogen under aerobic and anaerobic conditions, or in anaerobic-aerobic coupling systems [41]. The biodegradation period of dyes by the microorganisms depends on the growth rate and environmental conditions, and is directly related to the rate at which the wastes are metabolized. Therefore, kinetics is essential for the efficient design of reactors in the treatment of dye effluents [42,43].

Individual photocatalytic and microbiological processes are not as efficient if they are used individually for the transformation of recalcitrant pollutants or they lead to a mineralization process; therefore, combined processes are currently being studied that can increase this efficiency and, above all, eliminate the toxicity of some intermediary compounds. A combination of photocatalysis and biodegradation is a promising approach to the elimination of xenobiotic organic compounds from wastewater, since photocatalysis divides the molecules into simpler intermediates through free radical attack (hydroxyl radical, superoxide anions, and hydrogen peroxide) and easily generates biodegradable products that are then mineralized by microorganisms [44,45].

The results obtained in the waste characterization by FTIR after both processes showed the transformation of complex functional groups into simpler ones. Aromatic functional groups be transformed to the CH3 group in the range of 1023–1093 cm ^-1^ in Terasil Blue dye and 2850–3000 cm ^-1^ in Erionyl Blue dye [46–48], and the decrement of toxic-type azo groups in the range of 1634 cm ^-1^ [46,49], contributing to mineralization.

In addition to that above, the test conducted with *Daphnia magna* at the end of the combined degradation processes demonstrates, that there was no acute toxicity, being a fundamental parameter in this study, since wastewater can be used for crop watering.

## 5. Conclusion

The proposal of using the catalyst Pd/Al _80_ Ce _10_ Zr _10_ to degrade the 3 types of blue dyes in an aqueous medium showed that the effectiveness of the photocatalytic process depended on the molecular structure of the dye. There was sensitivity in the catalyst due to the incident of solar radiation, so simultaneous oxidation and reduction reactions that lead to a complex molecule destabilization of the dyes were promoted in the system. The treatment system proposed for the decolorization of the azo and anthraquinone dyes under analysis and formed by a (sunlight) photocatalytic-bacterial process worked properly by obtaining percentages higher than 87%, and proving the absence of toxicity in the final product. This can be considered an effective biotechnological process for the treatment of wastewater in the textile industry.
